# New fluorobenzamidine exerts antitumor activity against breast cancer in mice via pro-apoptotic activity

**DOI:** 10.1007/s12672-022-00554-6

**Published:** 2022-09-15

**Authors:** AbdelRahman B. Saleh, Nagwa H. Hassan, Mohamed A. Ismail, Wael M. El-Sayed

**Affiliations:** 1 Department of Zoology, Faculty of Science, Ain Shams University, Cairo, 11566 Egypt; 2 Department of Chemistry, Faculty of Science, Mansoura University, Mansoura, 35516 Egypt

**Keywords:** Bithiophene, Tamoxifen, Apoptosis, *CDK1*, *ESR*

## Abstract

**Background:**

Breast cancer is one of the leading causes of cancer-related morbidities. The present study aimed to evaluate the efficacy of bithiophene-fluorobenzamidine (BFB) against breast cancer induced by 7,12-dimethylbenz(a)anthracene (DMBA) in female Swiss mice and reveal the underlining mechanisms.

**Methods:**

The mice were randomly divided into five groups; control, BFB-treated group, DMBA-treated group, and the last two groups received DMBA then tamoxifen or BFB.

**Results:**

BFB reduced the tumor incidence by ~ 88% versus 30% after TAM. DMBA significantly increased the expression of *CDK1* and *HER2* and reduced the expression of *p53, p21 (CDKN1A)*, *ESR-α*, and *CAS3*. BFB caused significant down-regulation of *CDK1* and *HER2* and upregulation of *p53*, *p21*, *ESR-α,* and *CAS3*. In the DMBA-treated mice, cancerous cells metastasized to several organs. This was prevented by the administration of BFB. The antimetastatic and proapoptotic activities were confirmed in MCF7 cells in vitro by the wound healing and annexin V assays, respectively. Kaplan–Meier analysis showed that the BFB increased survival. In the DMBA group, tumors showed invasive carcinoma of grade III with central necrosis, polymorphism, mitotic activity, and numerous newly formed ductules, and colloidal mucinous secretions within adenoid cysts. BFB administration restored the normal structure of the mammary glands.

**Conclusion:**

Taken together, BFB has antitumor, pro-apoptotic, and anti-metastatic activities against breast cancer in mice and therefore, it merits further investigations.

**Supplementary Information:**

The online version contains supplementary material available at 10.1007/s12672-022-00554-6.

## Background

Breast carcinoma is the most prevalent malignancy among women worldwide. Over one million women are diagnosed with breast cancer annually. More than 40% of such patients die from complications, representing 14% of deaths from cancer in females [1]. Carcinogenesis results from excessive generation of oxidative free radicals, modifications in the DNA, and ultimately the loss of the usual regulatory pathways of cell differentiation, proliferation, and death [2]. Around 70% of the breast cancers express estrogen receptor alpha (ER-positive cells) [3]. Hormonal therapy is the most common treatment for ER-positive breast cancer patients. However, breast cancer cells have a high level of resistance and recurrence after hormonal therapy [4]. Tamoxifen (TAM) is a selective estrogen receptor regulator and is the most commonly used medication for the treatment of ER-positive breast cancer. TAM adjuvant treatment trials indicate a reduction in the risk of recurrence and decreased mortality by 40–50% [5]. However, de novo and acquired tamoxifen-resistance is the major constraint.

The advancement in the breast cancer treatment did not significantly reduce the annual mortality rate. Therefore, there is a greater need for more efficient and safe cancer prevention and treatment approaches. To achieve this goal, we need a reliable animal model for breast cancer. 7,12-Dimethylbenz[a]anthracene (DMBA) is one of the polycyclic aromatic hydrocarbons primarily emitted during the incomplete combustion processes [6]. Breast cancer induced by DMBA in a murine model has been frequently used for the development and assessment of chemo-preventive new medicines [7]. DMBA is usually given with a promoter such as progesterone. Progesterone promotes the cell proliferation by regulating the cell cycle genes, synthesis of growth factors, and receptors. Progestin (synthetic progesterone) activates angiogenic growth factors making the microenvironment suitable for tumor production and progression [8]. Progestins increase the proliferative capacity of latent breast tumor cells and/or facilitate tumor growth by activating and converting the dormant breast cancer stem cells into transitional subpopulations, which then differentiate into breast cancer cells [9]. Progestins have the ability to increase tumor cell proliferation after initiation, resulting in the appearance of palpable tumors earlier [10].

Cationic bithiophenes have been reported for their antimutagenic [11], and anticancer [12–14] activities. The present study developed a new protocol for breast cancer induction in normal Swiss albino mice, investigated the molecular mechanism(s) behind the DMBA-induced mammary carcinogenesis, and evaluated the efficacy of novel bithiophene fluorobenzamidine derivative (BFB) against DMBA-induced mammary tumors. BFB was tested by the National Cancer Institute (NCI) against a panel of 60 cancer cell lines. BFB had a median growth inhibition at 0.63 µM and was very safe having a median lethal concentration at ~ 49 µM. It was very effective against the growth of six breast cancer cell lines at 79 nM [11, 12]. These data were very promising and encouraged us to launch the present study.

## Materials and methods

### Chemicals

The DMBA was purchased from Sigma Aldrich Chemical Co. (Germany). The bithiophene fluorobenzamidine derivative (BFB) (See Additional file 1: Fig. S1A) was synthesized as previously described [11]. Medroxyprogesterone acetate (MPA, 150 mg/ml) and TAM (10 mg/Tab) were purchased from a local pharmacy (Cairo, Egypt). TRIzol for RNA extraction, and cDNA synthesis kits were purchased from Thermo Fisher Scientific Company (USA). The chemicals used in the histology study were purchased from AlGomhuria Company (Egypt). All chemicals were of analytical grade. MCF-7 cells were purchased from Nawah Scientific Company, Egypt. Cells were grown in DMEM medium (BioWhittaker™) supplemented with bovine serum albumin (10%, Life Science Group L, UK, Cat No: S-001B-BR) and with 100 IU/ml penicillin/streptomycin (100 µg/ml) (Lonza, 17-602E). BFB was prepared in dimethyl sulfoxide (10 mM stock) (Cat# 20385.02, Serva, Heidelberg, Germany) and stored at − 20 °C.

### Animals

A total of 140 virgin female Swiss albino mice (*Mus musculus*) at the age of 30 days weighing about 14–16 g were purchased from the VACSERA (Egypt). Mice were acclimated for 14 days at 22 °C ± 2 °C with a 12-h light/dark cycle. Animals were administered water and standard dried food ad libitum during the whole experiment. All animal experiments were carried out in accordance with the European Communities Council Directive of 24 November 1986 (86/609/EEC). The experiments were carried out at the animal facility of Department of Zoology, Faculty of Science, Ain Shams University (Egypt). All measures were taken to minimize any pain or suffering of the animals. All experiments were performed in accordance with ARRIVE guidelines. The study was approved by the Research Ethics Committee for animal research at the National Hepatology and Tropical Medicine Research Institute, research protocol No. 27-2019. The IACUC allowed larger tumor size (> 2 cm or > 20% of the body weight) due to the even time frame we asked for in all groups, but they asked for a tighter monitoring schedule for food and water intake, mobility, weight, activity, coat score, pain, distress, and chronic pain. The IACUC determined to humanely end the lives of two animals from the DMBA group and one from the DMBA/TAM that were in distress.

### Induction of breast cancer and TAM dose preparation

MPA vial (150 mg/ml) was diluted to a total volume of 4.5 ml then every animal was injected subcutaneously with 100 µl (3 doses/week for 4 weeks) which is equivalent to a total dose of 40 mg/animal. The DMBA (4 doses of 1 mg in 200 µl corn oil) was given after the MPA administration. TAM tablets were crushed into powder in foil paper to prevent photolysis, suspended in DMSO solution and stored in dark vials and administered at 5 mg/kg.

### Animals and experimental design

First, the LD_50_ of BFB administered orally in female mice was determined and found to be 25 mg/kg. After acclimatization, the animals were randomly divided into six groups and the whole experiment lasted for 42 weeks. Groups were divided as follows (See Additional file 1: Fig. S1B); group I (Control): 10 mice at the age of six weeks were left untreated for nine weeks then received 200 µl of 10% DMSO (p.o.) three times per week for 12 weeks. Group II (DMBA group, p.o.): 30 mice at the age of six weeks received MPA for 3 weeks then received MPA and DMBA for one week, then DMBA for three weeks (modified from Aldaz et al.) [15]. Group III (DMBA-group, i.p.): it was treated just like the second group, but the DMBA was injected i.p. Group IV (DMBA/BFB, p.o.): 30 mice received the same treatment as group II, left for a week to ensure DMBA metabolism. Then, they received three doses/week of 2.5 mg/kg (1/10 LD50) of BFB in 200 µl of 10% DMSO for 12 weeks. Group V (DMBA/TAM, p.o.): 30 mice were treated like group IV but received 5 mg/kg of TAM instead of BFB. Group VI (BFB control): 10 mice were treated with the BFB at 2.5 mg/kg in 200 µl of DMSO for 12 weeks.

### Body weight, tumor enumeration, and tissue collection

The body weight of mice was recorded on a weekly basis in all groups at the same time of the day. At the end of the experiment (42-weeks), the animals were anesthetized by isolflurane. The tumor incidence, multiplicity, numbers, and sizes were all calculated and recorded as described elsewhere [16]. A digital vernier calliper was used to determine the size of the tumors. Tumor incidence = (number of tumor bearing mice/total number of mice) × 100. Multiplicity = total number of tumors/total number of mice bearing tumors. Tumor size = Length/2 × Width/2 × π.

Mammary glands (control and BFB groups) or mammary tumors (from other groups) were collected, flushed with cold isotonic saline, and then separated into two sets. The first set was fixed in 10% formalin solution for 24 h at room temperature before staining with hematoxylin and eosin (H&E) (Sigma, USA). Slides were examined by light microscope (Olympus, Japan). The other set was immediately frozen using liquid nitrogen and stored in TRIzol (Thermofisher Scientific, USA) at − 80 °C for the q-RT PCR analysis.

### RNA extraction from mammary tissue, cDNA synthesis, and qRT-PCR

RNA was extracted from breast tissue and the purity of the extracted RNA was determined at 260/280 nm using UV-spectrophotometer (PG Instruments Limited, England). Using reverse transcription, 1 μg of RNA was converted to single-stranded complementary DNA (cDNA) by the High-Capacity cDNA Reverse Transcription Kit (Applied Biosystems, Life Technologies, USA). The Gene Amp PCR System 9700 from Applied Biosystems was used to make cDNA (Life Technologies, USA). Quantitative real-time PCR (qRT-PCR) was used to measure the relative expression levels of cyclin-dependent kinase 1 *(CDK1), p53,* cyclin dependent kinase inhibitor 1A *(p21* or *CDKN1A)* Estrogen receptor alpha *(ESR-α),* Receptor tyrosine-protein kinase *(Erbb2)* or *HER2,* and *Caspase-3 (CAS3)* genes in the mammary glands or mammary tumors. To assess the relative expression of the investigated genes, SYBR green master mix (Maxima SYBR Green/ROX qPCR Master Mix (K0251, Thermofisher Scientific, USA) was used. Glyceraldehyde 3-phosphate dehydrogenase (*GAPDH*) was used as housekeeping gene to normalize the mRNA expression. The thermal cycler (Agilent Stratagene MX3000P, USA) with optical 96-well plate and cycling conditions of (10 min/95 °C, followed by 40 cycles of 15 s/95 °C, 60 s/60 °C, and finally at 72 °C for 15 s) were used for qPCR techniques. The specific primers (See Additional file 1: Table S1) for the qRT-PCR were purchased from Invitrogen (Belgium) and used at a final concentration of 10 mM. The RQ = 2^−ΔΔCT^ equation was used to express the gene expression in relative units.

### Wound healing assay

The wound healing assay was performed to study the cell migration in vitro. A uniformed scratch was performed in a single monolayer, and the images were captured at zero time and at regular intervals thereafter. The scratch wound healing assay was performed with minor modifications as described elsewhere [17]. MCF7 cells were seeded in a 6-well plate (2.5 * 10^5^ cells/ml) and incubated overnight at 37ºC and 5% CO_2_. The medium was replaced by a fresh serum-free medium containing DMSO (0.01%) or BFB (at the IC_50_ = 1.06 µM). The scratch was produced using a sterile P200 tip. After 48 h, the cells were washed two times with ice-cold 1 × PBS and fixed with cold methanol for 30 min at RT. Cells were then stained with 0.5% crystal violet for 30 min. The extra stain was washed off with tap water. The images were taken using Optika B-159 (OPTIKA S.r.l., Italy). The size of the wound was measured using Image J 1.51 software. The migration rate was then calculated [(initial wound width − final wound width)/time].

### Apoptosis assay

MCF7 cells (1 * 10^5^ cells/ml) were seeded in a 6-well plate overnight as shown before. On the next day, the medium was replaced by fresh medium containing either DMSO (0.01%) or BFB (1.06 µM) and incubated for 48 h. The cells were trypsinized, harvested, and washed two times with 1×  PBS. The cells were stained with 5 µl Annexin-V-PE and 5 µl Propidium iodide (Elabscience Biotechnology Inc, E-CK-A211) in the binding buffer for 15 min. The stained cells were analyzed by using BD FACSCanto II™ Flow Cytometer.

### Statistical analysis

Statistical analysis was carried out using the Statistical Package for Social Science version 20 for computer windows (SPSS software package, Chicago, USA). The data distribution was tested by the Kolmogorov–Smirnov test. Data are presented as mean ± SD, with 95% confidence intervals calculated using nonlinear regression. The statistical analysis was performed using one-way ANOVA followed by Tukey’s test for multiple comparisons between the various groups and their respective controls, p values less than 0.05 were considered significant. Groups II and VI were compared with the control group (group I). Groups IV and V were compared with group II. The time of death of every mouse was recorded and analyzed using Kaplan–Meier survival statistics.

## Results

### Body weight gain and relative organ weight

**Fig. 1:**
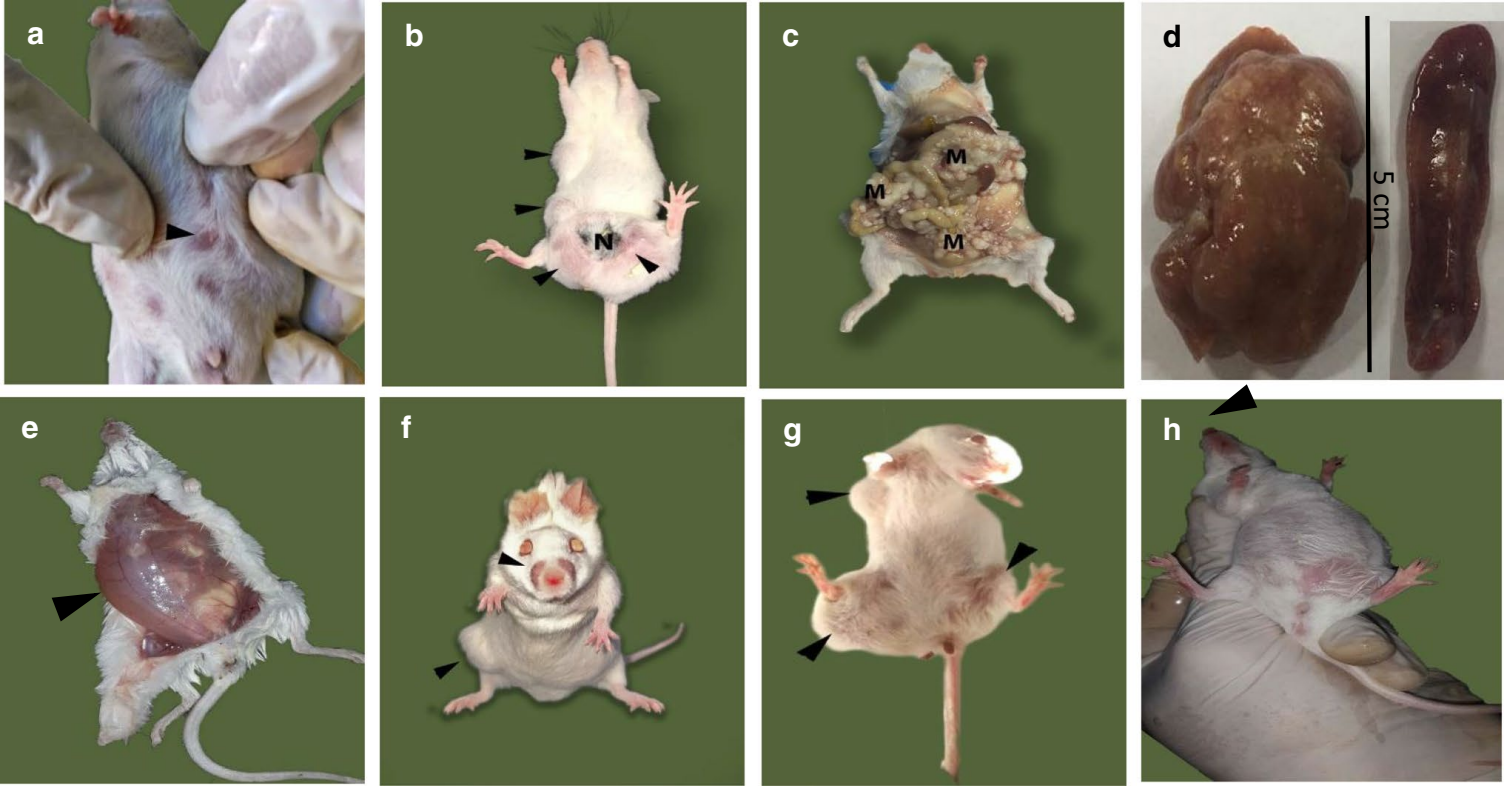
Effect on the macroscopic examination of animals. **a** Control group showing normal nibbles (black arrow). **b**–**d** DMBA (p.o.) group showing tumors (black arrows in b with central necrosis N), metastasis (M in **c**) and hepatosplenomegaly (**d**). **e** DMBA (i.p.) group showing fluid accumulation in peritoneal cavity. **f** DMBA/BFB group showing nasal hair loss and small tumor (black arrows). **g** DMBA/TAM group showing tumors (black arrows). **h** BFB group showing nasal hair loss (arrow)

DMBA injected i.p. caused the development of fluid accumulation at the peritoneal cavity and resulted in the death of all animals in group III (Fig. 1E). The administration of DMBA (p.o.) caused a significant reduction (− 41%) in the body weight gain, which was significantly reversed by BFB (+ 42% from the DMBA) while TAM did not cause any significant amelioration in the weight gain reduction caused by the DMBA (Table 1). BFB alone did not cause any significant weight change compared with the control. Although the DMBA decreased the body weight gain, it caused significant elevations in the relative weight of liver (+ 66%) and spleen (334%). BFB was able to reduce the weight of these organs compared with the DMBA group and almost restored the liver (+ 21% from control) and spleen (+ 13% from control) relative weights to the normal control levels, while TAM improved the spleen weight (+ 230% from control) without any significant change in the relative liver weight compared with the DMBA group (Table 1). Fatty liver changes were observed in the DMBA/TAM group.

**Table 1:** Effect of bithiophene-fluorobenzamidine (BFB) treatment on the body weight gain, and relative weights of liver and spleen

	Body weight gain (g)	Relative liver weight (%)	Relative spleen weight (%)
Control	12.8 ± 0.9	3.55 ± 0.17	0.53 ± 0.13
DMBA	7.6 ± 0.5^a^	5.88 ± 0.49^a^	2.30 ± 0.29^a^
DMBA/BFB	10.8 ± 0.9^b^	4.28 ± 0.57^b^	0.60 ± 0.08^b^
DMBA/TAM	8.4 ± 0.5	5.85 ± 1.05	1.75 ± 0.20^b^
BFB	11.5 ± 0.4	4.00 ± 0.38	0.63 ± 0.09

Results are expressed as Mean ± SD, n = 5

*DMBA* dimethylbenz(a)anthracene, *TAM* tamoxifen

^a^Significant versus control group

^b^Significant versus DMBA group

### Macroscopic examination of the animals, mortality, and tumor incidence, multiplicity, and size

The DMBA group showed many tumors with central necrosis (Fig. 1B) compared to normal appearance of the control (Fig. 1A). The breast cancer in some mice from the DMBA group metastasized to several body organs; lungs, intestinal tract, uterus, and ovaries (Fig. 1C). The mice from the DMBA and DMBA/TAM groups showed hepatosplenomegaly (Fig. 1D) consistent with the significant increase in the relative weights of liver and spleen reported in Table 2. The DMBA/BFB mice developed smaller and fewer number of tumors compared to the DMBA alone or even to the DMBA/TAM (Fig. 1G). In the DMBA/BFB and BFB groups, BFB administration induced full loss of nasal hairs in all mice of both groups (Fig. 1F and H, respectively).

**Table 2:** Effect of DMBA, TAM, and BFB on the mortality rate, and tumor incidence, multiplicity, count, and size

Groups	Mortality (%)	No. of animals examined	Tumor non-related mortality	Tumor related mortality	No. of tumors bearing animals	Total no. of tumors	Tumor incidence	Tumor multiplicity	Tumor size (cm^3^)
Control	10	9	1	0	0	0	0	0	0
DMBA (p.o.)	70	9	19	2	9	23	100	2.56	6.05 ± 0.19^a^
DMBA/BFB	43.3	17	13	0	2	3	11.7	1.50	1.46 ± 0.07^b^
DMBA/TAM	66.6	10	16	4	7	15	70	2.14	6.77 ± 0.17
BFB	30	7	3	0	0	0	0	0	0

Tumor incidence = number of tumor bearing mice/total number of mice. Multiplicity = total number of tumors/total number of mice bearing tumors

*DMBA* dimethylbenz(a)anthracene, *BFB* bithiophene-fluorobenzamidine, *TAM* tamoxifen

As shown in Table 3, BFB reduced the tumor incidence (from 100 to 11.7%), multiplicity (from 2.56 to 1.50), and number of total tumors (from 23 in 9 mice to 3 tumors in 17 mice), and average tumor size (from 6.05 to 1.46 cm^3^). TAM was much less effective (incidence 70%, multiplicity 2.14, total number of tumors 10 in 10 mice, and the average tumor size 6.77 cm^3^). The group administered with BFB only had a 30% mortality rate. The mice in the control and BFB groups did not show any spontaneous tumors (Table 2). BFB reduced the tumor-related death (death after the appearance of tumors) from 2 in the DMBA group to no deaths while TAM increased it to 4 deaths. The administration of BFB reduced the mortality rate from 70% in the DMBA group to 43.3%, while TAM reduced it to 66.6% (Table 2). The tumors in the DMBA and DMBA/TAM groups showed successive patterns of increase and decrease in size (See Additional file 1: Fig. S2A). As shown in the Kaplan Meier analysis, most of the mortalities occurred during the administration of DMBA. The mice in the DMBA/BFB and BFB groups did not suffer any death from week 18 to the end of 42 weeks. Some mice in the DMBA and DMBA/TAM groups died around weeks 33–37 (See Additional file 1: Fig. S2B).

**Table 3:** Summary of the histopathology changes in mammary gland and mammary tumors

Abnormalities	control	DMBA	DMBA/BFB	DMBA/TAM	BFB
Mammary tumors	-	++++	+	+++	-
Ductal carcinoma	-	+++	+	+++	-
Adenoid cyst carcinoma	-	+++	-	++++	-
Mitotic figures	-	++++	++	++++	+
Polymorphism	-	+++	-	+++	-
Vascular congestion	-	+	+	++	+
Intermammary lymph node involvement	-	+	+	++	+
Inflammatory cell infiltration	-	++	+	+++	+
Necrotic foci	-	++++	-	+++	-
Tumor grade	-	G3	G1	G2 and G3	-

- None, + mild lesions, ++ moderate lesions, +++ severe lesions, ++++ very severe lesions

*DMBA* dimethylbenz(a)anthracene, *BFB* bithiophene-fluorobenzamidine, *TAM* tamoxifen

### Gene expression pattern

**Fig. 2:**
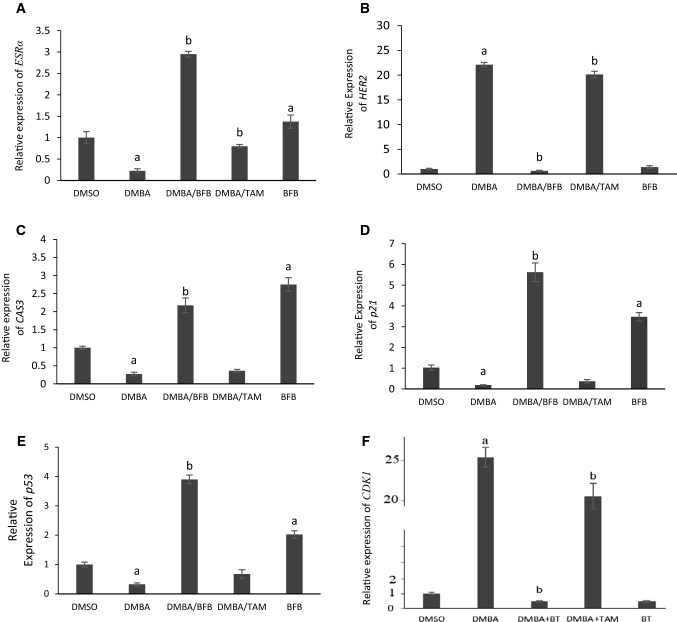
Relative Expression of **A** *ESRα* **B** *Erbb2 (HER2)* **C** *CAS3* **D** *CDKN1A (p21)* **E** *p53* and **F** *CDK1* genes in mammary tissue. Data are shown as Mean ± SD, n = 5. ^a^Significant vs. control, ^b^Significant vs. DMBA

DMBA caused a significant upregulation of *CDK1* and *HER2* by 26 and 22 fold, respectively (Fig. 2)*. p53*, *p21*, *ESR-α*, and *CAS3* were significantly downregulated by ~ 70, 80, 80, and 70%, respectively, in the DMBA group. The administration of BFB after DMBA exposure reversed the effect of DMBA and caused significant downregulation in the expression of *CDK1* and *HER2* and upregulations of *p53*, *p21*, *ESR-α* and *CAS3*. TAM administration caused a significant downregulation of *CDK1* and *HER2* compared to the DMBA group. The relative expression of *p53*, *p21* and *CAS3* were non-significantly upregulated after TAM in comparison to the DMBA group. The *ESR-α* was significantly upregulated after TAM. BFB alone given to healthy mice caused significant upregulation in *p53, p21, ESR-α,* and *CAS3* when compared to the control group (Fig. 2).

### Histological examination

**Fig. 3:**
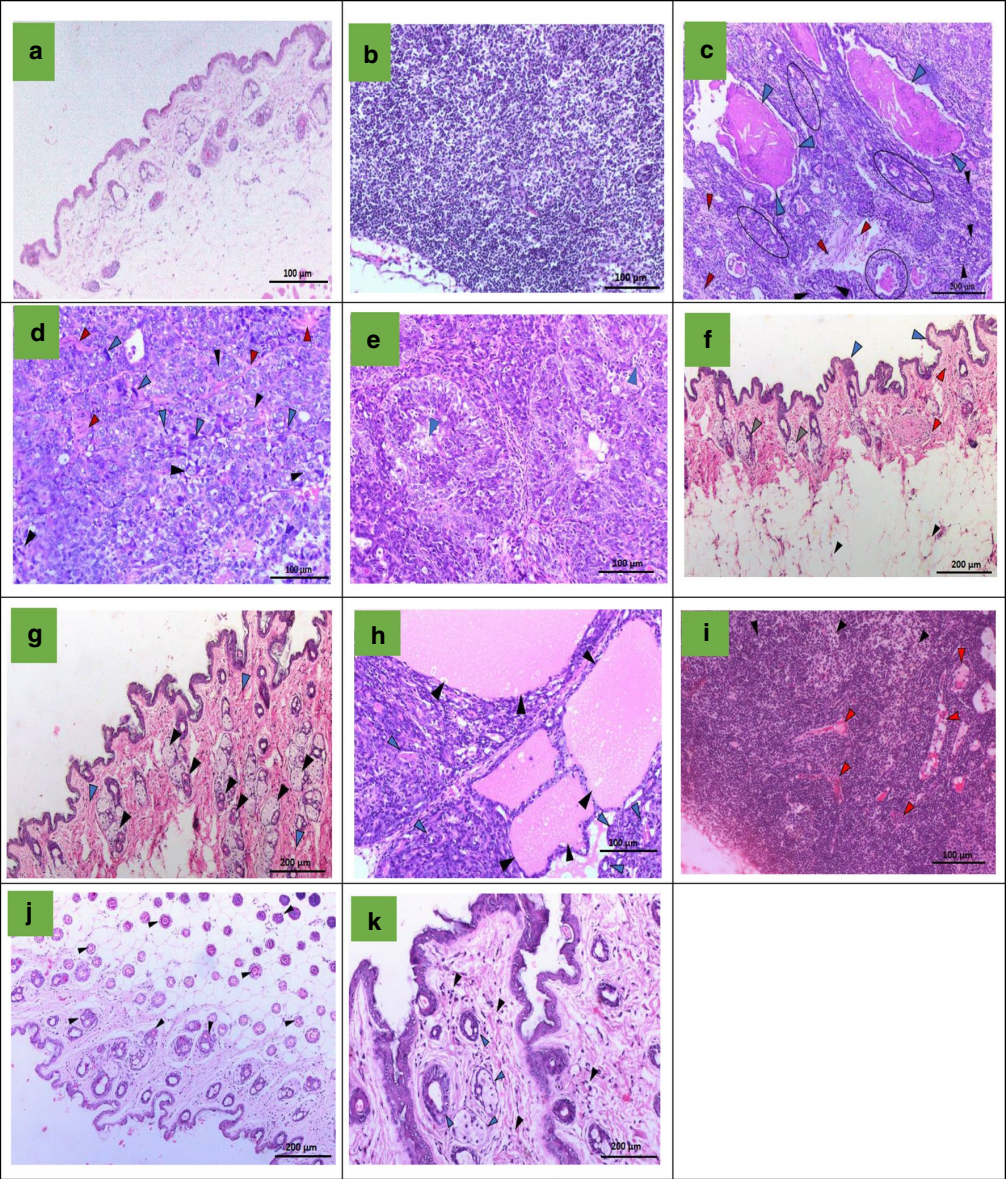
The histological examination of breast tissue. **a** and **b** Control showing normal skin including epidermis and dermis, normal acini in (**a**), and intact mammary lymph node (**b**). **c**–**e** The DMBA group (**c**) ductal carcinoma with colloidal secretion (blue arrows), newly formed ductules (oval shapes), moderate fibrosis (red arrows), proliferated wall duct (round shape), and invasive ductal carcinoma (black arrows), **d** Nuclear polymorphism (black arrows), mitotic activity (blue arrows), fibrosis (red arrows), and **e** necrosis (blue arrows). **f** and **g** DMBA/BFB, **f** normal epidermis (blue arrows), free acini (black arrows), dermis showing mild inflammation (red arrows), hyperplasia in hair follicles and sebaceous glands (gray arrows), **g** Hyperplasia in skin appendages including hair follicles and sebaceous glands (black arrows), mild inflammation (blue arrows). **h** and **i** DMBA/TAM, **h** ductal carcinoma and accumulation of colloidal secretion in ducts and acini (black arrows), newly formed ductules (blue arrows). **i** Hyperplasia of lymphoid follicles (black arrows) and congestion of blood vessels (red arrows). **j** and **k** BFB group, **j** normal epidermis, free acini, mild inflammation, hyperplasia in hair follicles and sebaceous glands and hair follicles in dermis (black arrows) and **k** hyperplasia in hair follicles and sebaceous glands (blue arrows), mild inflammation (black arrows)

Mice from the control group showed normal skin (epidermis and dermis) and acini (Fig. 3A), and mammary lymph nodes (Fig. 3B). DMBA resulted in numerous newly formed ductules formed due to the invasion of cancerous cells through the basement membrane of some breast ducts (Fig. 3C). Mild fibrosis was also seen in some areas between the mammary lobules (Fig. 3C). Adenoid cyst carcinoma could be seen in some areas in which colloid mucinous secretions accumulated in the wall of some proliferated ducts (Fig. 3C). DMBA induced the formation of ductal carcinoma where tumor cells showed polymorphism and mitotic activity (Fig. 3D). Tumors showed invasive type of grade III with areas of central necrosis and mild fibrosis (Fig. 3E). The administration of BFB reversed the effects of DMBA. The animals showed normal mammary glands with normal epidermis (Fig. 3F). The dermal layer showed hyperplasia in skin appendages including hair follicles and specious glands, activation of mononuclear/inflammatory cells, and mild vascular congestion that indicates mild inflammation (Fig. 3G). The intermammary lymph nodes showed mild hyperplasia. Most mice administered with TAM showed ductal carcinoma with the formation of newly formed ductules, mild fibrosis, polymorphism, mitotic activities, and cyst formation (Fig. 3H). Mice that did not show carcinoma showed inflammatory reactions detected around some ductules with congestion of the vasculature, and moderate hyperplasia was seen in the intramammary lymph nodes with moderate vascular congestion (Fig. 3I), inflammatory reactions could also be seen in the connective tissue of the dermal layer. BFB alone did not induce any deterioration in the structure of mammary glands; normal acini structures were seen. The dermal layer showed follicles in the deep dermis and acini (Fig. 3J). Epidermis of some individuals showed acanthosis in some areas together with infiltration of some mononuclear cells (Fig. 3K). The tumors in the DMBA group were from grade III which were also found in DMBA/TAM in addition to grade II tumors, while tumors of DMBA/BFB group were only at grade I (Table 3). In comparison to the DMBA and DMBA/TAM groups, BFB reduced the mitotic activities almost by half, stopped the appearance of any polymorphism and prevented the formation of any necrotic foci which were clearly seen in DMBA and DMBA/TAM groups (Table 3).

### Pro-apoptotic and anti-metastatic activities of bithiophene-fluorobenzamidine (BFB)

**Fig. 4:**
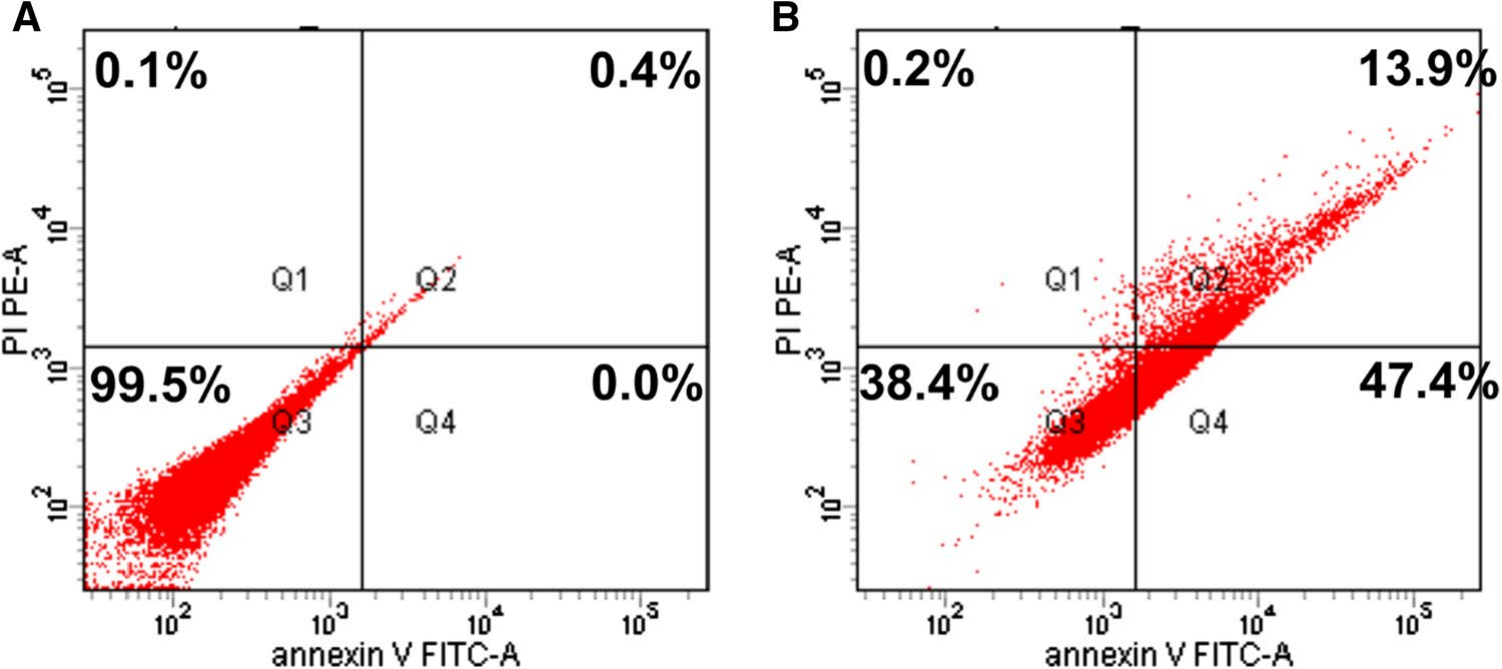
Induction of apoptosis in MCF-7 cells estimated by Annexin V. **A** Control, and **B** cells treated with BFB. Q1: necrotic cells, Q2: apoptotic cells, Q3: viable cells, Q4: Pro-apoptotic cells

**Fig. 5:**
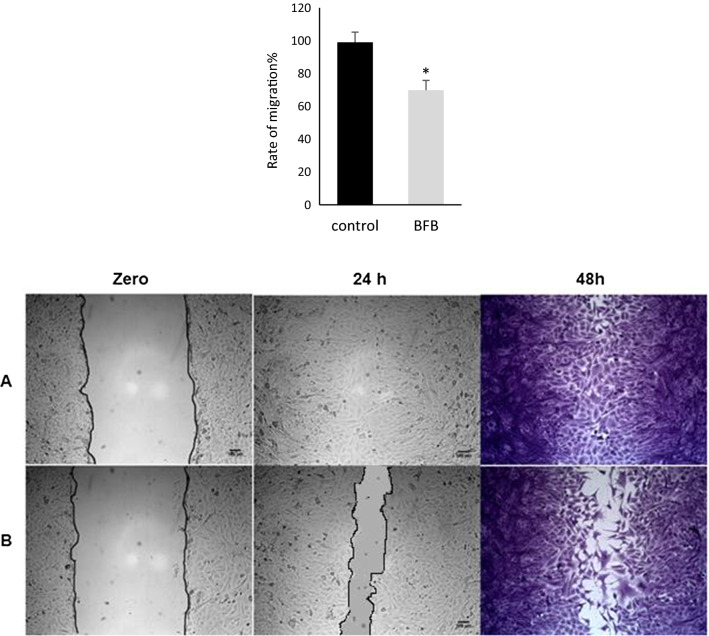
The wound healing assay in MCF-7 cells, **A** Untreated cells, **B** cells treated with bithiophene-fluorobenzamidine (BFB) at 1.06 µM (IC_50_). *Significant (p < 0.05) compared to untreated cells from two independent experiments

To confirm the pro-apoptotic activity, MCF-7 cells were treated with BFB for 48 h. BFB increased the early apoptotic cells from 0 to 47.4% and the late apoptotic cells from 0.4 to 13.9%. This means an increase in the total apoptotic cells from 0.4 to 61.3% or a 153-fold elevation in apoptosis in the breast adenocarcinoma cells. It is also noteworthy that necrotic cells increased only from 0.1 to 0.2% (Fig. 4). We have also performed a wound healing or scratch assay to assess the anti-metastatic activity of BFB in MCF-7 cells. BFB caused a ~ 29% decline in the migration rate compared to the untreated cells but only after 48 h (Fig. 5).

## Discussion

The intraperitoneal administration of DMBA resulted in the death of all animals before the development of tumors. DMBA caused a significant decrease in body weight like what was reported in previous studies [18, 19]. DMBA induces the generation of reactive oxygen species and lipid peroxidation (LPO) in the cellular membrane, and this was reported to reduce body weight [20]. Although it reduced body weight gain, DMBA caused a significant elevation in the relative liver and spleen weights. The liver is the primary site for the activation and metabolism of DMBA [21]. The treatment with BFB was able to alleviate the deleterious effects of DMBA on the body weight and the relative weight of liver and spleen. This alleviation may be explained by the antiproliferative and antioxidant effects of BFB previously reported [11, 12, 14]. TAM was much less effective than BFB. The reduction in both tumor and non-tumor related deaths caused by BFB indicated the superior efficacy of BFB over TAM. The use of TAM is associated with many side effects, which include an elevated risk of endometrial cancer, cataracts, pulmonary embolism, and thrombosis [22].

The DMBA-treated groups showed 100% incidence of breast cancer and the tumors metastasized to several body organs such as lung, intestinal tract, uterus, and ovary in accordance with previous studies [23, 24]. Treatment with BFB prevented metastasis to other organs which can be explained by the downregulation of *HER2* reported in the current study. This was also supported by the effect of BFB on MCF-7 cells, where BFB reduced the migration of cells by ~ 29%. Upregulation of *HER2* is thought to be responsible for the cancer cell survival and metastasis [25]. In the Kaplan–Meier analysis, the mortality was mainly distributed around the interval of DMBA administration and the period following it, which reflects the severe toxic effect of DMBA. BFB caused a significant reduction in the mortality greater than that observed after the administration of TAM.

BFB reduced the tumor incidence (by ~ 88%), multiplicity (by 41%), and tumor size (by ~ 76%) as well as the total number of tumors when compared to the DMBA group. BFB showed less toxic and more ameliorative effects than TAM. TAM reduced the incidence and multiplicity by 30 and 16%, respectively. However, TAM increased the tumor size by 12%. BFB was previously reported to have the same effects on the colorectal polyps induced by dimethyl hydrazine in rats [26] and on acute leukemia in mice (unpublished data). BFB was also previously shown to have antiproliferative activity against a panel of 60 cancer cell lines with median GI50 value at 0.63 µM. This panel included six breast cancer cell lines with individual GI50 values at 0.079–1.52 µM [12]. In the current study, BFB significantly elevated p53 expression, an effect that was previously reported to reduce the tumor volume [27].

Breast cancer occurs when genetic changes in proto-oncogenes, tumor suppressors, or repair genes occur [28]. The proto-oncogenes that contribute to the development of breast tumors include C-myc, Neu/HER2 and Cyclin D whereas p53 is the major tumor suppressor gene [29]. DMBA causes severe damage to organs rich in lipid content such as breast and bone marrow due to its high solubility in lipids. Unlike lymphoid organs, the mammary gland accumulates DMBA and maintains adequate levels for several days after a single 30 mg dose and acts as a DMBA reservoir [30]. DMBA causes the induction of mammary tumors as a result of covalent binding of DMBA metabolites such as DMBA-3,4-dihydrodiol-1,2-epoxide to the DNA of breast cells forming DNA-adducts causing mutations in several critical genes [31]. DMBA also depletes the antioxidant enzymes through the massive oxidative stress exerted by free radical production that cause LPO and initiation of carcinogenesis [32]. The antioxidant activity of the BFB was previously reported [14, 26].

*p53* plays a crucial role in controlling the progression of the cell cycle, apoptosis, and stability of DNA. Any DNA damage results in the increase of the expression of *p53* which stops the cell cycle at the G1/S checkpoint triggering DNA repair mechanisms [33]. If the damage is severe, *p53* initiates apoptosis [34] through stimulating p21 [35]. This will result in the suppression of cell growth in cancerous cells [36]. In the current study, the elevation in *p53* may be responsible for the corresponding increase in *CAS3* expression; the principal executing component responsible for apoptosis [37]. The ability of the cell to proceed to phase S depends on *CDK1* [38]. The downregulation of *CDK1* by BFB, along with the increase in the expression of *p53*, indicates arrest of the cell cycle. DMBA reduced apoptosis as indicated by the downregulation of *CAS3* and *p53* in agreement with a previous study [39]. BFB increased apoptosis by upregulating *p53*, *CAS3*, and *p21*, and inhibits *CDK1* causing cell cycle arrest [40]. It also increased apoptosis in MCF-7 cells which express estrogen and progesterone receptors by 153-fold. These cells were selected due to the resemblance of biochemical characteristics they have to the tumors formed after DMBA.

Estrogen receptors such as *ESR-α* are specific proteins bound to estrogen within the breast cells. Numerous cells of breast cancer have receptors which bind to the estrogen hence the name ER+ tumors [41]. Binding estrogen to the estrogen receptor induces phosphorylation of the receptor causing dimerization of the receptor and promotes binding of the receptor complex to the target gene promoter area for transcription activation [42]. The estrogen-bound ER conformation promotes the recruitment of coactivators which increase the activity of transcription causing increased cell survival and proliferation [43]. Conversely, the ER conformation caused by TAM binding facilitates the recruiting of corepressors that suppress transcriptional activity. ER in or near the plasma membrane can stimulate epidermal growth factor receptor (*EGFR)* and *HER2/neu*, which are growth factor tyrosine kinases that provide a further pathway for the growth-promoting influence of estrogen [44]. The over-expression of *ESR-α* and *EGFR* signaling pathways are associated with mammary carcinoma [45]. The kinase cascade induced by the EGFR family members such as *HER2* turns on the ER and its co-regulatory proteins, resulting in a vicious cycle leading to increased tumor cell proliferation and survival [46]. In another study, TAM was shown to be an estrogen agonist in breast cancer cells with high *HER2* levels, resulting in tamoxifen-resistance and enhancement of mammary tumor growth [47].

DMBA administration resulted in the formation of *HER2*+ mammary tumors which express excessive amounts of HER2 protein. *HER2* is frequently amplified during the development of mammary tumors [48]. Mammary tumors which over-express the *HER2* gene are characterized by rapid growth, development and TAM-resistance [49]. In the DMBA/TAM group, there was minimal ameliorative effect of TAM that blocks the ESR-α receptor, which can lead to the occurrence of TAM-resistance. The development of breast tumors that express *ESR-α* is accelerated by the presence of estrogen, which in turn facilitates the endocrine treatment strategies, whereas breast tumors not expressing *ESR-α* show primary endocrine-treatment resistance [50]. The existence of *ESR-α* therefore coincides with improved disease-free survival and more effective prognosis in contrast with ESR-α negative breast tumors [51].

In the present study, DMBA induced the induction of mammary tumors with significant downregulation of *ESR-α.* This downregulation is responsible for the development of resistance to treatment [52]. BFB administration caused significant upregulation in the *ESR-α* gene in DMBA/BFB and BFB groups that may restore the sensitivity to hormonal treatment. It has been reported that the treatment of ESR-negative breast cancer cells with hypomethylating agents results in the re-expression of active *ESR* [53]. Such results suggest the investigation of the concomitant use of the BFB along with TAM in treating the TAM-resistant breast cancer.

Mice from the DMBA group showed ductal carcinoma with polymorphism and mitotic activity. Tumors showed invasive type of grade III with central necrosis and mild fibrosis as previously reported [54]. Tumors increased in size through the formation of numerous newly formed ductules that were penetrated by mild fibrosis. The administration of BFB reduced the tumor grade to I, incidence of ductal carcinoma, mitotic activity, polymorphism and prevented the formation of any cyst carcinoma or necrotic foci, which indicates a decrease in tumor volume. The mild ameliorative effect of TAM administration may indicate the occurrence of TAM-resistance which was reflected by the appearance of advanced mammary tumors of grade II to III with adenoid cyst carcinoma, polymorphism, and mitotic activity confirming the results of the gene expression. The DMBA-treated group showed significant increase in the tumor volume. In the present study, fluctuations in the tumor size over time in the same mice have been reported. Cycles of increases and decreases over time in the DMBA-treated group could represent cycles of cell proliferation and death. The accelerated growth of tumor cells could ultimately exceed the rate of angiogenesis resulting in hypoxia [55] accompanied by the death of cancer cells in many areas and the subsequent emergence of necrotic areas inside the tumor that is confirmed by the histopathological examination (Fig. 3E). This could cause inflammatory reactions due to the release of endogenous molecules known as damage-associated molecular patterns (DAMPs). DAMPs and macrophages have powerful pro-angiogenic properties [56] that could contribute to a corresponding increase in tumor volume (Fig. 3C). In the DMBA-TAM group, the liver showed fatty changes, which did not appear in any other group treated with DMBA. BFB caused hyperplasia in hair follicles and sebaceous glands, an effect that deserves further investigations.

## Conclusions

In the present study, DMBA induced mammary tumors through upregulation of *CDK1* and *HER2,* and downregulation of *p53*, *p21*, *ESR-α*, and* CAS3*. BFB has antitumor activity through the direct interaction with several genes and reversed all the previous effects of DMBA on the genes investigated. BFB showed superior efficacy over TAM in enhancing survival, and reducing the tumor size, incidence, and multiplicity. BFB also improved the mammary gland architecture and almost normalized the histopathology caused by the DMBA. Monotherapy with selective estrogen receptor regulator such as TAM has little or only modest obvious benefits on *ESR-* and *HER2*-over-expressing mammary tumors. Taken together, BFB has antitumor, pro-apoptotic, and anti-metastatic activities against breast cancer in mice and MCF-7 cells in vitro and, therefore, it merits further investigations.

## Supplementary Information

**Additional file 1: Table S1. **Primers used for RT-PCR analysis. **Figure S1. A** Structure of bithiophene derivative (BFB) [11]. **B** Schematic diagram for experimental design. **Figure S2. A** The tumor size development through 12 weeks from the time of appearance of the first tumor till the end of experiment. **B** The Kaplan Meier analysis of survival.

## Data Availability

All data presented in this manuscript are reported in the manuscript and additional files.
