# Supplementary material for: New fluorobenzamidine exerts antitumor activity against breast cancer in mice via pro-apoptotic activity
Source: Discov Oncol. 2022 Sep 15;13:88. doi: 10.1007/s12672-022-00554-6 (PMC9478011; doi:10.1007/s12672-022-00554-6)
Supplement: Supplementary file 1 — Additional file 1: Table S1. Primers used for RT-PCR analysis. Figure S1. A Structure of bithiophene derivative (BFB) [11]. B Schematic diagram for experimental design. Figure S2. A The tumor size development through 12 weeks from the time of appearance of the first tumor till the end of experiment. B The Kaplan Meier analysis of survival. [file 12672_2022_554_MOESM1_ESM.docx]

New **Fluorobenzamidine** Exerts Antitumor Activity against Breast Cancer in Mice via Pro-apoptotic Activity

AbdelRahman B. Saleh^1^, Nagwa H. Hassan^1^, Mohamed A. Ismail^2^, Wael M. El-Sayed^1,*^

**Table S1: Primers used for RT-PCR analysis**

| Gene | Forward | Reverse |
| --- | --- | --- |
| *GAPDH* | GCATCTTGGGCTACACTGAG | TATTCATTGTCATACCAGGAAATG |
| *CDK1* | AAATATAGTCAGCCTGCAGGAT | AGTAACTCTTAACGAGTGAAGAAT |
| *p53* | AAACTACTTCCTCCAGAAGATAT | GCTTCACTTGGGCCTTCAAA |
| *p21* | GTACTTCCTCTGCCCTGCT | TTGGAGTGATAGAAATCTGTCA |
| *ESR-α* | TAACGAGAAAGGAAACATGATCAT | TCATTGTGTCCTTGAATGCTTCT |
| *HER2* | TCAGCCTCAAAACAGCTCG | TTCGTCCAGGTCCACACATG |
| *CAS3* | TCATCTCGCTCTGGTACGGA | TGTGTAAGGATGCGGACTGC |


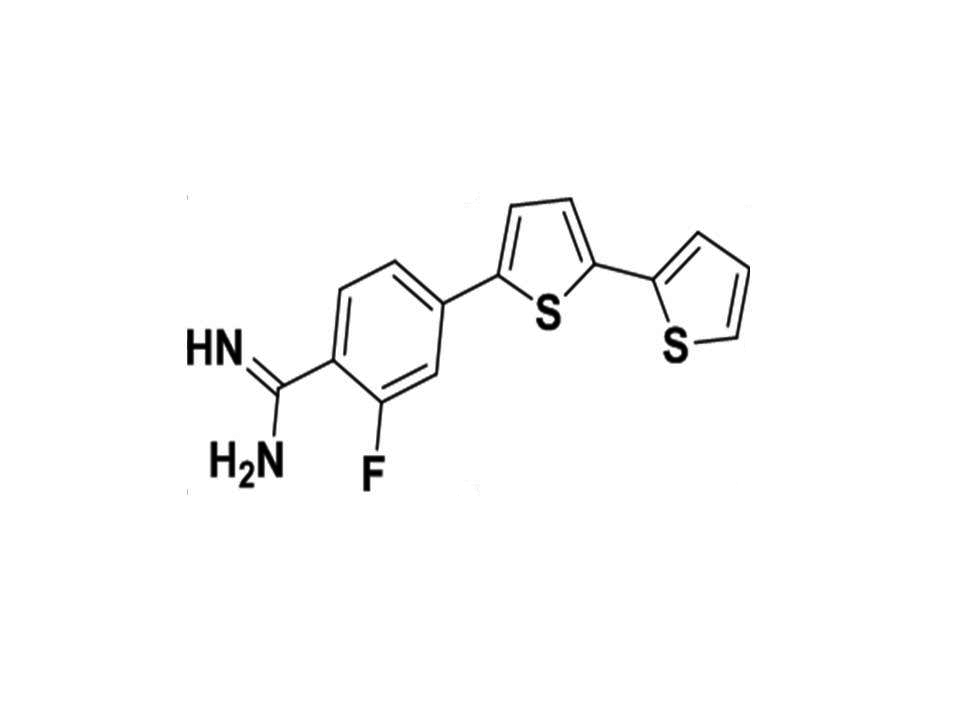


**Figure S1A:** Structure of bithiophene derivative (BFB) **[11]**


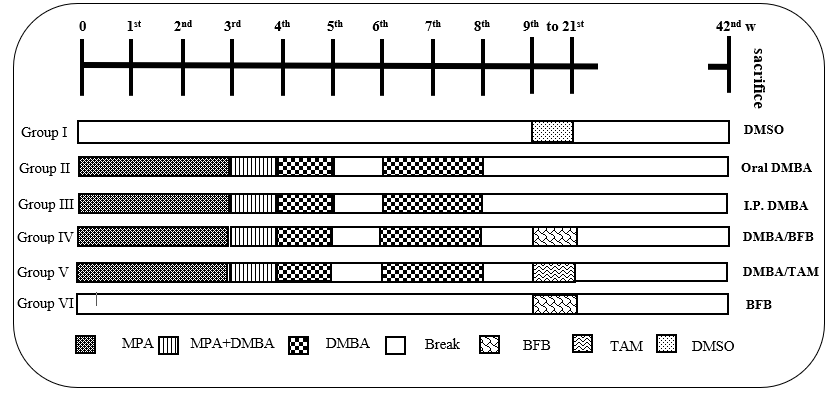


**Figure S1B:** Schematic diagram for experimental design


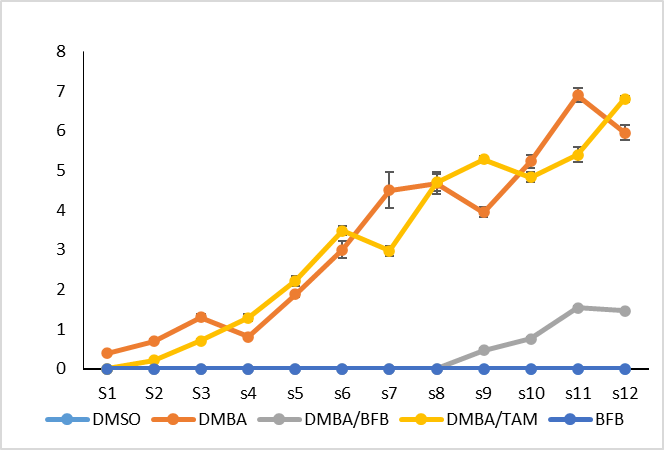


**A**

Tumor Size (cm)


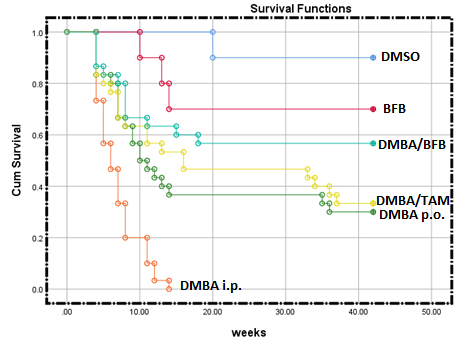


**B**

**Figure S2:** A) The tumor size development through 12 weeks from the time of appearance of the first tumor till the end of experiment. B) The Kaplan Meier analysis of survival
